# AFTER THE STORM: SOCIAL WORK STUDENTS ASSISTING WITH FEMA DISASTER RELIEF FOR OLDER ADULTS

**DOI:** 10.1093/geroni/igac059.1718

**Published:** 2022-12-20

**Authors:** Joy Ernst

**Affiliations:** Wayne State University, Detroit, Michigan, United States

## Abstract

This poster describes a partnership during Fall 2021 between the Wayne State School of Social Work and the Federal Emergency Management Agency (FEMA) to assist older adults whose homes sustained damage during flooding in June 2021. This short-term project helped people access resources to repair and restore damaged homes, repair, or replace mechanical and electrical systems, deal with mold and other health hazards, and replace their personal belongings. Social work students trained by FEMA made over 700 outreach calls to homeowners whose applications for relief were rejected. The students worked with the homeowners to facilitate access to help that either resulted in the approval of their FEMA application (due to assistance with technical issues such as missing documentation or errors in the application) or connected them with alternative sources of help and support. A focus group with students provided insights on their motivations, training experiences, issues affecting the applicants, and skills developed. While FEMA-required training offered little relevant assistance, SSW staff and faculty overseeing the program provided ongoing support that students considered vital as they worked to assist applicants. Some homes were extensively damaged and resources available from FEMA were insufficient. Some homeowners suspected the students were scammers; students also learned of exploitation by contractors. Students honed empathy, reflection, and supportive listening skills as they heard stressors associated with maintaining their homes amidst losses due to covid and their knowledge of community resources expanded. Lessons learned to aid in future efforts to assist in disaster relief are described.

